# Effect of Sodium-Glucose Co-transporter 2 (SGLT2) Inhibitors in the Management of Hyponatremia Associated With Syndrome of Inappropriate Antidiuresis

**DOI:** 10.7759/cureus.97037

**Published:** 2025-11-17

**Authors:** Yasir Kammawal, Zaheer Ahmad Kammawal, Tajdar Ali, Shahzad Ahmad, Wiqar Ahmad

**Affiliations:** 1 Internal Medicine, St. Mary's Hospital, Isle of Wight NHS Trust, Newport, GBR; 2 Internal Medicine, Ulster Hospital - South Eastern Health and Social Care Trust, Belfast, IRL; 3 Internal Medicine, Northwest General Hospital and Research Centre, Peshawar, PAK; 4 Medicine, Northwest General Hospital and Research Centre, Peshawar, PAK

**Keywords:** hyponatremia, osmotic diuresis, sglt2 inhibitors, siad, siadh

## Abstract

Background: Hyponatremia due to syndrome of inappropriate antidiuresis (SIAD) is a common and challenging electrolyte disorder, particularly in hospitalized and elderly patients. Conventional treatments, such as fluid restriction and vasopressin receptor antagonists, have limitations related to efficacy, tolerance, availability, and cost. Sodium-glucose co-transporter 2 inhibitors (SGLT2i), primarily used for type 2 diabetes, have shown potential in promoting osmotic diuresis and free water clearance, making them a promising therapeutic alternative for SIAD-induced hyponatremia.

Methodology: A retrospective cohort study was conducted at Northwest General Hospital, Peshawar, Pakistan, analyzing medical records of 50 adult patients diagnosed with SIAD-associated hyponatremia between March and August 2025. Patients who received SGLT2i during treatment were included. Data on demographics, laboratory values (including serum sodium levels), and treatment outcomes were collected. The primary outcome was the change in serum sodium after SGLT2i initiation. Statistical analyses were performed using paired t-tests, with p < 0.05 considered significant.

Results: The mean baseline serum sodium was 122.4 ± 4.8 mmol/L. Within 72 hours of starting SGLT2i therapy, the mean sodium level increased to 129.1 ± 3.9 mmol/L (p < 0.001), with 72% of patients achieving normonatremia (≥135 mmol/L) within one week. The mean sodium increase at 72 hours was +6.7 ± 2.8 mmol/L. Mild, self-limited polyuria occurred in 16% of patients. No serious adverse events, volume depletion, or renal function deterioration were observed.

Conclusion: SGLT2i significantly improved serum sodium levels in patients with SIAD-associated hyponatremia, demonstrating a favorable safety and tolerability profile. These findings suggest that SGLT2i may offer a viable, accessible alternative to traditional therapies for managing hyponatremia in SIAD. Prospective randomized trials are needed to confirm these results and establish long-term safety and efficacy.

## Introduction

The most frequent electrolyte imbalance seen in clinical practice is hyponatremia, which is characterized by a blood sodium content of less than 135 mmol/L and is linked to higher rates of morbidity and mortality [1]. One of the most common causes of hyponatremia, especially in hospitalized and elderly patients, is the syndrome of inappropriate antidiuresis (SIAD), formerly known as the syndrome of inappropriate antidiuretic hormone secretion (SIADH) [2]. Despite normal or low plasma osmolality, SIAD is characterized by the non-osmotic production of arginine vasopressin (AVP), which results in concentrated urine, dilutional hyponatremia, and decreased free water excretion [3].

Historically, fluid restriction, salt pills, loop diuretics, and, more recently, vasopressin receptor antagonists (vaptans) have been used to treat SIAD-induced hyponatremia [4]. These treatments do have certain drawbacks, though. While vaptans are costly, need close monitoring because of the possibility of an excessively quick correction of serum sodium, and are not generally accessible in many areas, fluid restriction is frequently poorly tolerated and inconsistently successful [5]. Therefore, new, secure, and efficient therapeutic approaches for SIAD-associated hyponatremia are constantly needed.

The pleiotropic effects of sodium-glucose co-transporter 2 inhibitors (SGLT2i), which were initially created as antihyperglycemic medications for the treatment of type 2 diabetes mellitus, have drawn attention. These consist of natriuretic qualities, renal advantages, and cardiovascular protection [6]. SGLT2i work by blocking the proximal renal tubule's ability to reabsorb glucose and salt. This causes osmotic diuresis, glucosuria, and mild natriuresis, all of which are potentially helpful in treating hyponatremia and water retention [7].

According to new research, SGLT2i may facilitate free water clearance through a different pharmacologic pathway, much like vaptans do, in a manner akin to aquaresis [8]. SGLT2i may offer an alternate or supplemental method of treating hypo-osmolar conditions such as SIAD-induced hyponatremia by causing osmotic diuresis through glucosuria, particularly in patients who are unable to follow fluid restriction or who are intolerant to vaptans [9].

Serum sodium levels in individuals with SIAD or chronic hyponatremia have improved after starting SGLT2i, according to preliminary clinical investigations and case reports, especially in those who also had diabetes or heart failure [10]. However, there are not many reliable prospective data. Thus, a comprehensive assessment of the safety and effectiveness of SGLT2i in this particular population is still required. The extent of serum sodium correction, the improvement's time course, and the possibility of too quick correction or volume depletion are still significant problems.

Investigating how SGLT2 inhibitors affect serum sodium levels in patients with SIAD-associated hyponatremia is the goal of this study. We intend to assess this possible therapeutic role in order to guide clinical judgment and investigate the repurposing of an existing class of drugs to treat an electrolyte condition that is prevalent and frequently challenging to treat.

## Materials and methods

Study design and setting

The Department of Medicine at Northwest General Hospital and Research Centre in Peshawar, Pakistan, was the site of this retrospective cohort study. To evaluate the impact of SGLT2i on hyponatremia linked to SIAD, medical records of patients treated over the six months before the study's start were examined.

Ethical approval

On September 29, 2025, the Institutional Evaluation Board (IRB) of Northwest General Hospital and Research Centre granted ethical permission for the retrospective evaluation of patient records (permission number: IRB&EC/2025-GH/0302). Because the study was retrospective in nature and patient data were anonymized, informed consent was not required.

Study population

Medical records of adult patients (≥18 years) diagnosed with SIAD between March 2025 to August 2025 were screened. Patients who received SGLT2i as part of their management for SIAD-associated hyponatremia were included. Patients with incomplete medical records, those with other causes of hyponatremia (e.g., adrenal insufficiency or hypothyroidism), or those on SGLT2i for diabetes mellitus prior to SIAD diagnosis were excluded.

Data collection

Data were extracted from hospital electronic medical records and patient charts, including demographic details, clinical history, laboratory parameters (serum sodium, plasma osmolality, urine sodium, urine osmolality), treatment details, and outcomes. Serum sodium levels at diagnosis and during follow-up visits were recorded to evaluate treatment efficacy.

Pharmacological treatment protocol

SGLT2i were used as an adjuvant therapy in addition to the traditional management of SIAD, which usually involved correcting any identifiable underlying reasons and limiting fluid intake to 800-1,000 mL per day. SGLT2i utilized during the trial period were empagliflozin and dapagliflozin, which were chosen based on drug availability and physician judgment. Dapagliflozin was provided at a dose of 10 mg once daily, and empagliflozin was started at a dose of 10 mg once daily, both taken orally in the morning. Since these conventional therapeutic doses were utilized off-label to treat hyponatremia linked to SIAD, no dose escalation was carried out. Depending on the clinical response and the return of serum sodium levels to normal, the course of treatment could last anywhere from seven to 28 days. Once serum sodium stabilized within the normal reference range (135-145 mmol/L) or if any negative side effects were noted, therapy was stopped. Serum sodium, plasma and urine osmolality, urine sodium, renal function parameters (serum creatinine and estimated glomerular filtration rate), and clinical volume status were all serially assessed throughout baseline and follow-up intervals (days 3, 7, 14, and 28, where available). During SGLT2 inhibitor therapy, none of the patients received tolvaptan or hypertonic saline concurrently, and loop diuretics were not administered unless necessary to treat comorbid volume overload. Through examination of patient charts, adverse events that may be connected to the use of SGLT2i were meticulously recorded, including hypotension, dehydration, urinary tract infection, and acute renal injury.

Outcome measures

The primary outcome was the change in serum sodium concentration following initiation of SGLT2 inhibitor therapy. Secondary outcomes included time to normalization of sodium levels, adverse events, and the relationship between sodium correction and clinical characteristics.

Statistical analysis

Data were analyzed using Statistical Product and Service Solutions (SPSS, version 26.0; IBM SPSS Statistics for Windows, Armonk, NY). Continuous variables were expressed as mean ± standard deviation or median (interquartile range), depending on data distribution. Categorical variables were expressed as counts and percentages. Paired t-tests or Wilcoxon signed-rank tests were used to compare pre- and post-treatment sodium levels. A p-value < 0.05 was considered statistically significant.

## Results

A total of 50 patient records diagnosed with SIAD and treated with SGLT2i between March 2025 and August 2025 were included in the analysis. The mean age of patients was 54.3 ± 12.7 years, with a male predominance (60%, n=30). Baseline characteristics are summarized in Table 1.

**Table 1:** Baseline Characteristics of the Study Participants (n = 50)

Variable	Value
Age (years), mean ± SD	54.3 ± 12.7
Gender, n (%)	
Male	30 (60%)
Female	20 (40%)
Baseline serum sodium (mmol/L), mean ± SD	122.4 ± 4.8
Baseline plasma osmolality (mOsm/kg), mean ± SD	260 ± 10
Baseline urine sodium (mmol/L), mean ± SD	45 ± 12
Baseline urine osmolality (mOsm/kg), mean ± SD	520 ± 100

Following initiation of SGLT2i therapy, serum sodium levels showed a significant increase at 72 hours post-treatment (mean: 129.1 ± 3.9 mmol/L), with normalization (serum sodium: ≥135 mmol/L) achieved in 36 patients (72%) within one week of treatment. The mean change in serum sodium from baseline to 72 hours was +6.7 ± 2.8 mmol/L (p < 0.001) (Table 2).

**Table 2:** Serum sodium levels before and after SGLT2 inhibitor treatment

Timepoint	Serum Sodium (mmol/L), mean ± SD	p-value*
Baseline	122.4 ± 4.8	
72 hours post-treatment	129.1 ± 3.9	<0.001
7 days post-treatment	134.7 ± 3.5	<0.001

No serious adverse effects related to SGLT2i were documented. Mild polyuria was reported in eight patients (16%) and resolved without intervention. There were no instances of volume depletion or worsening renal function.

Subgroup analysis revealed no significant difference in sodium correction between males and females (p = 0.45) or among different age groups (p = 0.33).

## Discussion

This retrospective investigation showed that SGLT2i significantly improved serum sodium levels in individuals with SIAD-associated hyponatremia, indicating their potential as a successful treatment option. The average rise in serum sodium was clinically significant and in line with new research that indicates SGLT2i's osmotic diuretic and natriuretic actions can ameliorate hyponatremia and address water retention [11,12]. These results are consistent with earlier case reports and smaller studies showing that SGLT2i may address the pathophysiology of SIAD by improving free water clearance without causing excessive sodium loss, hence restoring sodium balance [13,14].

The main cause of hyponatremia in SIAD is improper production of antidiuretic hormones, which results in dilutional hyponatremia and water retention [15]. Traditional therapies, such as hydration restriction and demeclocycline, have drawbacks such as nephrotoxicity and low adherence, respectively [16]. Despite their demonstrated effectiveness, vasopressin receptor antagonists are frequently expensive and not generally accessible [17]. As similarly described by Salvatore et al. [18] and Packer [19], our data support the growing importance of SGLT2i as a more readily available and well-tolerated option that offers efficient sodium correction without major side effects.

SGLT2i work by preventing the proximal renal tubule from reabsorbing glucose and sodium, which causes osmotic diuresis and increases the excretion of free water [20]. Because of this mechanism, they are especially well-suited for SIAD, where the main problem is excessive water retention. Crucially, neither volume depletion nor severe renal impairment was seen in our group, indicating a favourable safety profile in this population that is in line with the results of recent clinical trials in patients with diabetic nephropathy and heart failure [21,22].

The quick adjustment of sodium levels within 72 hours is encouraging and comparable to findings from prospective pilot studies by Fenske et al. [23] and Nigro et al. [24], despite the fact that our study was retrospective and had no control group. Improvements in patient symptoms and shorter hospital stays were also reported in these studies. However, given that chronic hyponatremia can lead to cognitive impairment and increased morbidity, longer-term trials are required to assess sustained efficacy and safety. Our results showed no discernible age or sex-based disparities in treatment response, indicating that SGLT2i may be widely applicable to a variety of patient populations. Large-scale meta-analyses in related conditions that show persistent benefits irrespective of demographic considerations corroborate this conclusion. Furthermore, no patients stopped treatment because of side effects, and the most frequent side effect was minor polyuria, which is to be expected given that SGLT2i are diuretics.

To validate these findings and compare SGLT2i to other proven treatments for SIAD, prospective randomized controlled trials are necessary in the future. To maximize salt correction and patient outcomes, research should also investigate combination strategies incorporating SGLT2i and fluid restriction or vasopressin antagonists. Furthermore, in order to better understand the renal and systemic effects of SGLT2i in SIAD beyond their glucose-lowering capabilities, mechanistic research is required

Limitations

There are a number of limitations to this study that should be noted. Because selection biases and unmeasured variables may have affected the observed results, the retrospective cohort design makes it difficult to determine a clear causal link between the use of SGLT2i and changes in serum sodium levels. Furthermore, the lack of a comparison group undergoing normal therapy, such as vaptans or fluid restriction alone, limits the ability to make direct clinical comparisons and weakens the conclusions. The results' applicability to larger or more varied clinical populations may be further limited by the single-center context and very small sample size. Additionally, potentially significant subgroups were not represented by removing individuals who were already on SGLT2i for diabetes mellitus, which could result in selection bias. Finally, insufficient data collection for certain clinical parameters may have resulted from variations in follow-up time and dependence on pre-existing medical records. To confirm these results and elucidate the specific therapeutic role of SGLT2i in SIAD-related hyponatremia, future multicenter, prospective, randomized controlled studies with bigger and more diverse populations, uniform follow-up, and predetermined objectives are necessary.

## Conclusions

In conclusion, our research bolsters the growing body of evidence that SGLT2i are a safe and efficient treatment option for treating hyponatremia in SIAD patients. SGLT2i provide a novel way to treat dilutional hyponatremia without causing serious side effects by increasing free water clearance and osmotic diuresis. SGLT2i may prove to be a useful addition to the treatment toolkit for this difficult clinical condition because of their accessibility and tolerability.
